# Time Spent Working in Custody Influences Work Sample Test Battery Performance of Deputy Sheriffs Compared to Recruits

**DOI:** 10.3390/ijerph16071108

**Published:** 2019-03-28

**Authors:** Robert G. Lockie, Robin M. Orr, Matthew R. Moreno, J. Jay Dawes, Joseph M. Dulla

**Affiliations:** 1 Department of Kinesiology, California State University, Fullerton, CA 92831, USA; moreno.matthewr@csu.fullerton.edu; 2 Tactical Research Unit, Bond University, Robina, QLD 4229, Australia; rorr@bond.edu.au; 3 Department of Health Sciences, University of Colorado-Colorado Springs, Colorado Springs, CO 80918, USA; jdawes@uccs.edu; 4 Recruit Training Unit, Training Bureau, Los Angeles County Sheriff’s Department, Los Angeles, CA 90022, USA; JMDulla@lasd.org

**Keywords:** aerobic fitness, body drag, fence climb, foot pursuit, job-specific, law enforcement officer, obstacle course, police, tactical

## Abstract

This study determined the influence of years spent working in custody on fitness measured by a state-specific testing battery (Work Sample Test Battery; WSTB) in deputy sheriffs. Retrospective analysis was conducted on one patrol school class (51 males, 13 females) divided into three groups depending on time spent working in custody: DS24 (<24 months; *n* = 20); DS2547 (25–47 months; *n* = 23); and DS48+ (≥48 months; *n* = 21). These groups were compared to a recruit class (REC; 219 males, 34 females) in the WSTB, which comprised five tasks completed for time: 99-yard (90.53-m) obstacle course (99OC); 165-pound (75-kg) dummy drag; six-foot (1.83-m) chain link fence (CLF) and solid wall (SW) climb; and 500-yard (457.2-m) run (500R). A univariate analysis of covariance (ANCOVA) (controlling for sex and age) with Bonferroni post hoc determined significant between-group differences. DS48+ were slower in the 99OC compared to the REC (*p* = 0.007) and performed the CLF and SW slower than all groups (*p* ≤ 0.012). DS24, DS2547, and DS48+ were all slower than REC in the 500R (*p* ≤ 0.002). Physical training should be implemented to maintain fitness and job-specific task performance in deputy sheriffs working custody, especially considering the sedentary nature of this work.

## 1. Introduction

Law enforcement can be a physically demanding profession; during a shift, on-duty officers may be required to drive vehicles [1], pursue suspects [2,3,4], clear obstacles [5], discharge firearms [6,7], and exert force and apprehend offenders [5,7,8,9]. Recruits must undergo specific training before they can become law enforcement officers (LEOs). The academy setting is where recruits are trained to tolerate the physical rigors of the profession while also learning the required procedures and skills necessary for policing [5,10,11,12]. As a result, most agencies have set standards that must be achieved by recruits when they are tested in job-specific tasks and physical fitness [2,12]. Failure to achieve these standards will generally mean a recruit will be separated from their academy and they will not graduate to become an LEO [12].

One example of a job-specific examination of physical skills is the Work Sample Test Battery (WSTB), which must be completed by law enforcement recruits before they graduate from law enforcement academies in California, USA [5,13]. As described by Lockie et al. [5], the WSTB consists of five tests that are completed for time: a run around a 99-yard (90.53-m) obstacle course (99OC); a body drag (BD) with a 165-pound (74.84-kg) dummy; a climb over a six-foot chain link fence (CLF); a climb over a six-foot solid wall (SW); and a 500-yard (457.2-m) run (500R). According to guidelines set by California’s Peace Officer Standards and Training (POST), these job-specific tests must be completed within specific time limits, which results in points being allocated to each task [13]. A minimum score of 384 in the WSTB is required. Recruits attain points within each task relative to their time to perform each task [5,13]. A faster task completion results in a greater point allocation [5,13].

Despite the importance of the WSTB being an indication of job task performance for recruits [13], once they graduate, they no longer have to maintain any performance standard. Indeed, few law enforcement agencies (LEAs) mandate that their LEOs meet standards of physical fitness over the course of their career [14,15]. As a result of this, many LEOs experience a decline in physical fitness over time. Although this could be related to age-related declines in physical performance [16,17], there are other factors that can also contribute. Orr et al. [15] compared the physical fitness of police academy cadets (recruits in the context of this study) and incumbent officers. The results indicated that male cadets were superior in muscular endurance (measured by push-ups and sit-ups completed in 60 s) and aerobic fitness (measured by 2.4 km run time) when compared to the aged-matched incumbent LEOs. The female cadets were superior in maximal strength (measured by a one-repetition maximum bench press) and upper-body endurance (push-ups completed in 60 s) compared to the female LEOs. Orr et al. [15] suggested the nature of law enforcement occupations, which feature a low volume of work-related physical activity, significantly impacted the maintenance of fitness in the LEOs. Given that superior strength, endurance, and aerobic capacity have been related to better performance in the WSTB [5], any declines in these qualities could negatively impact job performance, as indicated by tests such as the WSTB.

Decreases in fitness and job-specific task performance could be exacerbated for LEOs who spend extended periods of time in an environment where the job demands are lower than those encountered when on patrol, such as when working in custody. Custody facilities can include jails, detention, or court lockup facilities [18]. Although LEOs working in custody may be required to respond to emergencies and physically confront and restrain inmates, the predominant job tasks are low-intensity. This includes processing and supervising inmates, office work, and cell searches [18,19,20]. In accordance with these expected lower intensity job conditions, agencies that hire individuals for custody-only positions (e.g., custody assistants) generally do not mandate the testing of physical fitness in these individuals [18,21,22]. As custody assistants are a non-warranted position who work under the directives of sworn personnel [18], LEOs generally need to work in custody facilities as well. For deputy sheriffs from certain agencies in the USA, their first position after graduating academy is working in custody. Deputy sheriffs may spend several months to several years working in custody, depending on whether there are available patrol positions at the station they are assigned to. The length of time they spend in custody could influence their fitness and ability to complete job-specific tasks, which is an issue if they move onto patrol duties after working custody. However, there has been no analysis of the effects that time spent completing custody work could have on the fitness of deputy sheriffs.

Therefore, a cross-sectional analysis of deputy sheriffs from one patrol school class was conducted to analyze the influence of time spent working in custody on WSTB performance and compared to established WSTB standards for recruits from this LEA [5]. As the WSTB is used as an indicator for job preparedness in deputy sheriffs [5,13], it is assumed that if WSTB performance is maintained, the deputy sheriffs would be more physically prepared for the demands of patrol. It was hypothesized that the recruits would be faster in the WSTB tasks when compared to the deputy sheriffs. It was further hypothesized that deputy sheriffs who had spent more time working in custody would also be slower in the WSTB tasks.

## 2. Materials and Methods 

### 2.1. Subjects

Retrospective analysis on one patrol school class from one LEA comprised of 64 deputy sheriffs (age: 32.28 ± 6.54 years; height: 1.76 ± 0.08 m; body mass: 86.02 ± 16.10 kg), which included 51 males (age: 30.92 ± 5.74 years; height: 1.75 ± 0.07 m; body mass: 90.98 ± 13.76 kg) and 13 females (age: 37.62 ± 6.96 years; height: 1.61 ± 0.03 m; body mass: 66.57 ± 7.56 kg), was conducted. All deputy sheriffs from this patrol school class completed the WSTB, and only data from their time in patrol school were provided by the LEA to the researchers. Additionally, only the WSTB times for the deputy sheriffs were provided to the researchers, and not what would be the derived point allocation. The deputy sheriff data were compared to recruits from four academy classes from the same agency who successfully passed the WSTB. The data from these recruits have been previously published by Lockie et al. [5]. The recruit sample was comprised of 253 recruits (age: 26.69 ± 5.26 years; height: 1.75 ± 0.10 m; body mass: 79.69 ± 12.29 kg); 219 males (age: 26.69 ± 5.35 years; height: 1.77 ± 0.08 m; body mass: 81.94 ± 10.98 kg) and 34 females (age: 26.68 ± 4.68 years; height: 1.62 ± 0.09 m; body mass: 64.43 ± 9.57 kg). All class cohorts started their academy within a calendar year in southern California, USA. Based on the retrospective nature of this analysis, the institutional ethics committee approved the use of pre-existing data (HSR-17-18-370).

### 2.2. Procedures

The data in this study were collected by staff working for one LEA. The staff were all trained by a certified Tactical Strength and Conditioning Facilitator who verified the proficiency of the staff members. Age, height, body mass, and time spent working in custody were recorded at the start of patrol school. Patrol school was a three-week skills refresher program completed by incumbents who had been working in custody, as they did not complete any patrol duties during this time. Deputy sheriffs self-reported the time they had spent working in custody. For the recruits, age, height, and body mass were recorded at the start of the 22-week academy training period [5]. For the deputy sheriffs and recruits, height was measured barefoot using a portable stadiometer (Seca, Hamburg, Germany), while body mass was recorded by electronic digital scales (Health o Meter, Neosho, Missouri, USA). The deputy sheriffs completed the WSTB within the first week of patrol school, between 0700–1200 (7:00 a.m.–12:00 p.m.). As noted by Lockie et al. [5], the WSTB for the recruits was completed during the final weeks of academy depending on the class schedule, and typically between 0500–1200 (5:00 a.m.–12:00 p.m.). The weather conditions for testing for both patrol school and the academy classes were typical of the climate of southern California during a calendar year. Although conducting testing outdoors is not ideal, there was no indoor testing facility available for this LEA, and these procedures were typical of staff from the LEA [5,12,18,21,22,23,24]. 

### 2.3. Work Sample Test Battery (WSTB)

As noted, the WSTB is mandatory for LEAs in California, and recruits must attain a certain standard (minimum score of 384) in order to graduate from academy [13]. The focus of this study was the time required to complete each task in the WSTB rather than the point allocation. The procedures for each assessment have been presented by Peace Officer Standards and Training [13] and previously published by Lockie et al. [5] but are documented here as well. All tests were performed outdoors on structures specifically designed for the LEA training facility. Deputy sheriffs and recruits wore physical training attire (i.e., no equipment) during completion of the WSTB. The order of test completion may have varied between deputy sheriffs and recruits depending on time constraints, but the 500R was always completed last. This follows stated guidelines from POST, whereby WSTB events can be completed in any order as long as the 500R is the final event [13]. Deputy sheriffs and recruits were provided the opportunity for two attempts for each test (with a minimum of two minutes rest between attempts). Time was recorded to the nearest 0.1 s by a handheld stopwatch for each attempt, and the fastest time was recorded. Timing via stopwatches is standard practice in LEA testing [5,9,10,12,17,18,25,26,27]. Furthermore, testers trained in the use of stopwatch timing procedures for running and exercise tests can record reliable data [28]. 

**Figure 1:**
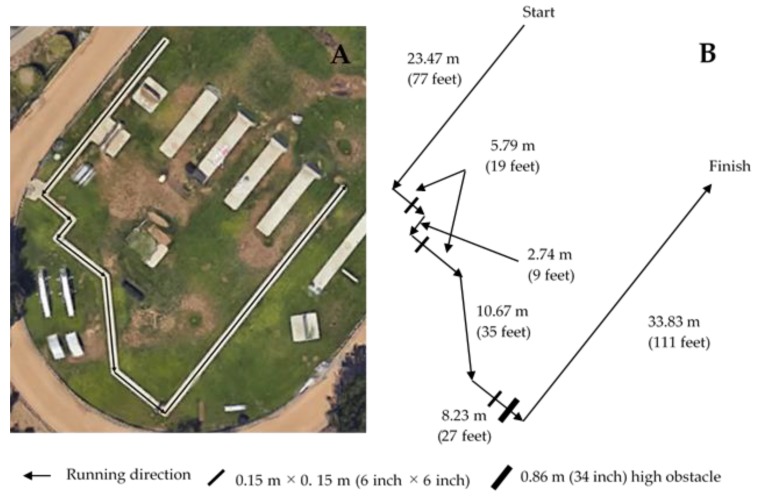
The 99-yard obstacle course. (**A**) Aerial map of the course. (**B**) Dimensions and running direction.

99-yard obstacle course run (99OC): This test was designed to simulate a foot pursuit and is shown in Figure 1. The 99-yard (90.53-m) course was completed as quickly as possible, and deputy sheriffs and recruits were to remain on the concrete track throughout the course. During the run, individuals also needed to step over three 6-inch × 6-inch (0.15 × 0.15 m) simulated curbs and one 34-inch (0.86-m) high obstacle.

Body drag (BD): Deputy sheriffs and recruits were required to drag a 165-pound (74.84-kg) dummy a distance of 32 feet (9.75 m). Individuals needed to pick up the dummy by wrapping their arms underneath the arms of the dummy and lifting it to a standing position by extending the hips and knees. Once the individual was standing with the dummy and they informed the tester they were ready, timing was initiated, and the individuals had to drag the dummy as quickly as possible by walking backwards over the required distance.

Chain link fence climb (CLF): Deputy sheriffs and recruits started 5 yards (4.57 m) away from the fence, and once the test was initiated, they were required to run up to and scale the fence with whatever technique they deemed most appropriate. However, they could not use any side supports on the fence to assist their climb. If the individual did not initially climb the fence in their first attempt within a trial, they could continue attempting to climb, but the time continued to run within the test. Once the individual cleared the fence, they were to land and run 25 yards (22.86 m) as fast as possible to complete the test.

Solid wall fence climb (SW): The same instructions and procedures for the CLF were provided for the SW, with the only difference being the type of wall that needed to be climbed.

500 yard run (500R): This test simulated a long-distance foot pursuit. The 500-yard (457.2-m) distance was marked on an athletics track, and deputy sheriffs and recruits were instructed to cover this distance as quickly as possible.

### 2.4. Statistical Analysis

All statistical analyses were computed using the Statistics Package for Social Sciences (Version 25.0; IBM Corporation, New York, NY, USA). Descriptive statistics (mean ± SD) were calculated for each test parameter. The sample was divided into four groups: REC (recruits; *n* = 253); DS24 (deputy sheriffs who worked in custody for ≤24 months; *n* = 20); DS2547 (deputy sheriffs who worked in custody for 25–47 months; *n* = 23); and DS48+ (deputy sheriffs who worked in custody for ≥48 months; *n* = 21). These time periods were selected to provide a relatively equitable group distribution across the deputy sheriffs. Levene’s test for equality of variances was used to ascertain the homogeneity of variance for the data, with significance set as *p* < 0.05. If data were found to be heterogeneous, the alpha level required for between-group significant interactions was adjusted to *p* < 0.01 to reduce the chance of making a Type I error. A univariate analysis of covariance (ANCOVA) was used to ascertain whether there were significant differences between the groups. The ANCOVA analysis was utilized due to the robustness of these procedures when used with a large sample, even with unequal group sizes [29,30]. Sexes were combined within each of the groups, as there are no corrections for sex in the WSTB [13]. Nonetheless, sex was used as a covariate, as numerous studies have documented sex differences in the physical performance of law enforcement populations [16,18,23,24,31]. Body mass and WSTB data were also analyzed with age as an additional covariate, as age can influence body mass and fitness test performance [15,16,17]. If a significant interaction between the groups was found, a Bonferroni post hoc adjustment for multiple pairwise comparisons was adopted (*p* < 0.05). Similar to previous research [12,23], effect sizes (*d*) were also calculated for the between-group comparisons, where the difference between the means was divided by the pooled SD [32]. A *d* less than 0.2 was considered a trivial effect; 0.2 to 0.6 a small effect; 0.6 to 1.2 a moderate effect; 1.2 to 2.0 a large effect; 2.0 to 4.0 a very large effect; and 4.0 and above an extremely large effect [33]. Differences between the mean times for the WSTB tasks through the time points established with the REC and deputy sheriff groups (REC were considered the baseline or 0 months; DS24: up to 24 months working in custody; DS2547: 25–47 months working in custody; DS48+: 48 months or greater working in custody) were also calculated. This was done to ascertain whether there was a certain time point where a clear decline in performance of a WSTB task could be established so as to provide support to findings from the ANCOVA.

## 3. Results

Descriptive data for the WSTB for all groups are shown in Table 1, while the effect size data are shown in Table 2. Homogenous data were indicated for age (F_3_ = 1.297, *p* = 0.276), height (F_3_ = 1.297, *p* = 0.133), body mass (F_3_ = 0.747, *p* = 0.525), 99OC (F_3_ = 0.630, *p* = 0.596), and the BD (F_3_ = 0.472, *p* = 0.702). The alpha level for significance for these data was set to *p* < 0.05. Heterogeneous data were indicated for the CLF (F_3_ = 14.615, *p* < 0.001), SW (F_3_ = 9.612, *p* < 0.001), and the 500R (F_3_ = 10.668, *p* < 0.001). The alpha level for significance for these data was set to *p* < 0.01. 

There was a significant interaction for age (F_3_ = 22.535, *p* = 0.006). REC was younger than all other groups (*p* ≤ 0.020; moderate-to-large effects); DS24 and DS2547 were younger than DS48+ (*p* ≤ 0.007; moderate effects). There was no significant between-group interactions for height (F_3_ = 22.535, *p* = 0.584). When controlling for sex and age, there was a significant interaction for body mass (F_3_ = 4.870, *p* = 0.003). However, the post hoc analysis indicated that even though there was a moderate effect for the difference between REC and DS48, this comparison did not achieve significance (*p* = 0.059). There was a significant interaction for the 99OC (F_3_ = 3.880, *p* = 0.010), with DS48+ being significantly slower than REC (*p* = 0.007), which had a moderate effect. There were also moderate effects for the faster 99OC times for DS24 and DS2547 compared to DS48+, but neither achieved significance (*p* = 0.410 and 0.437, respectively). There was no significant interaction for the BD (F_3_ = 0.464, *p* = 0.708). There was a significant interaction for the CLF (F_3_ = 6.408, *p* < 0.001) and SW (F_3_ = 5.547, *p* = 0.001). DS48+ performed both the CLF (*p* ≤ 0.004; all moderate effects) and SW (*p* ≤ 0.012; moderate effects for comparisons with REC and DS24) significantly slower than all other groups. There was a significant interaction for the 500R (F_3_ = 19.649, *p* < 0.001), with all deputy sheriff groups being significantly slower than REC (*p* ≤ 0.002; moderate-to-large effects).

**Figure 2:**
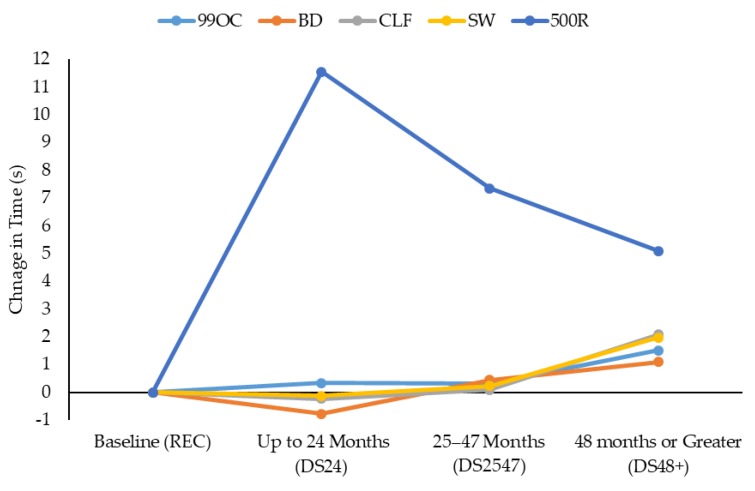
Difference in mean times for each WSTB task (99OC, BD, CLF, SW, and 500R) through the time points established in this study with the deputy sheriff groups (REC: baseline; DS24: up to 24 months working in custody; DS2547: 25–47 months working in custody; DS48+ 48 months or greater working in custody).

Figure 2 displays the difference in the mean times for the WSTB tasks through the time points established with the deputy sheriff groups (REC: baseline; DS24: up to 24 months working in custody; DS2547: 25–47 months working in custody; DS48+: 48 months or greater working in custody). For the 99OC, BD, CLF, and SW, there was a notable change in task performance past 48 months of custody work (which, as stated, was significantly different from the baseline for the 99OC, CLF, and SW; Table 1). For the 500R, the most pronounced change in performance time came after 24 months, before this was reduced in subsequent time points. Nonetheless, all of these were significantly different from the baseline established by the REC (Table 1).

## 4. Discussion

This study analyzed the influence of time spent working in custody on a state-specific job-specific testing battery called the WSTB in deputy sheriffs. It was hypothesized that a longer time spent working in custody would result in poorer WSTB performance in deputy sheriffs when compared to recruits and other deputy sheriffs who had spent less time working in custody facilities. The results provided support to these hypotheses. The DS48+ group performed poorer in the 99OC, CLF, SW, and 500C compared to the REC, and in the CLF and SW compared to the other deputy sheriff groups as well. All deputy sheriff groups were slower in the 500R compared to the REC. In support of previous research [15], these results suggest that LEAs should implement some form of physical training programs for their incumbent deputy sheriffs to maintain their fitness and job-specific skills. This is potentially even more important for deputy sheriffs who have to spend time working in custody before they move onto patrol duties. This is because the general duties of custody can be relatively low-intensity [20], especially compared to those that can be required on patrol (e.g., pursuing and restraining offenders, urgent driving, obstacle clearance) [34,35].

As expected, there were significant differences in age between the REC and deputy sheriff recruits. The mean age for the REC was typical for this agency [5,12,23,24,26,36], and it would be expected that the deputy sheriffs would be older, as they are several months or years into their career. The height and body mass of the REC was also typical for this agency [5,12,23,24,26,36]. Body mass tends to increase in males and females from their 20’s to 30’s [37]. However, in this study, there were no significant differences between the groups in body mass. Body mass is important to monitor in LEOs and deputy sheriffs, as increased body mass and fat can be indicative of increased cardiovascular disease risk [38,39,40,41], which is a major health issue in law enforcement populations [42,43,44]. Numerous authors have recommended that LEOs (inclusive of deputy sheriffs) should maintain physical activity across their career to ensure better health and fitness [15,25,42]. As is discussed in this paper, this could impact their performance of job-specific tasks as well.

The 99OC is designed to simulate a foot pursuit within an urban area [13]. Lockie et al. [5] detailed that the 99OC had small correlations with fitness tests such as push-ups, sit-ups, mountain climbers, pull-ups, and a 201-m run in recruits. All of these tests place some emphasis on anaerobic energy systems [45], which indicates the importance of these qualities in the performance of the 99OC, and by extension, a foot pursuit. The results from this study showed that DS48+ were slower in the 99OC compared to the REC. It would be expected that the REC would be faster in the 99OC, as they were at the end of their 22-week academy designed to specifically train them for policing duties [5]. For those deputy sheriffs who had spent 48 months or longer working in custody, the combination of the demands of law enforcement shift work (e.g., irregular and long hours) [15,46] with the low-intensity demands of custody work [18,19,20] likely contributed to the decline in physical characteristics contributing to a slower 99OC. Given that the DS48+ were moving to a patrol position, this would suggest that some form of physical training should be completed during the custody period to minimize any declines in fitness that could influence the performance of a job-specific task, such as a foot pursuit. Rossomanno et al. [25] found that a six-month supervised exercise program for LEOs that incorporated aerobic training and calisthenics could improve time to complete an obstacle course incorporating specific policing skills (e.g., running, jumping, obstacle clearance, dummy drag, and shooting). The LEA from this study should also consider implementing this type of program, especially for deputy sheriffs who need to work a patrol position after custody.

The 500R is also a maximum running task designed to simulate foot pursuit; in this instance, one conducted over a longer distance [13]. Similar to the 99OC, Lockie et al. [5] found that the 500R had small-to-moderate correlations with fitness tests such as push-ups, sit-ups, mountain climbers, pull-ups, and a 201-m run in recruits. In addition, due to the duration of the run placing demands on the aerobic system [45], the 500R also had a large correlation with the 2.4-km run. However, in contrast to the 99OC, all deputy sheriff recruits performed the 500R slower than the REC. This drop in performance was notable from the first time point established in this study (up to 24 months), as opposed to the 99OC, where the significant drop in performance only occurred past 48 months. These data suggest the decline in physiological characteristics important for the 500R (e.g., aerobic capacity) occurs early during a deputy sheriff’s custody tenure. As aerobic fitness training tends to be emphasized in LEA academy training [5,12,47,48], it would be expected that REC would be faster in the 500R. Additionally, Orr et al. [15] documented that the incumbent male LEOs were slower in the 2.4-km run compared to cadets, while Lockie et al. [17] found 2.4-km run times tended to get slower with advancing age in incumbent male officers. Given the relationship between the 500R and 2.4-km run [5], if performance in the 2.4-km run declines, it could be surmised that the 500R would decline also. As for the 99OC, if the 500R is viewed as being indicative of a foot pursuit, deputy sheriffs who come from working in custody should attempt to maintain their high-intensity running capacity and aerobic fitness before beginning their patrol tenure. This should occur either at the individual-level or with the provision of structured physical training from the LEA.

Lockie et al. [5] stated that the CLF and SW provide a test of how well a recruit can scale an obstacle such as that they might encounter in urban areas. Upper-body strength measured by pull-ups and muscular endurance measured by push-ups and sit-ups completed in 60 s have been found to relate to faster CLF and SW performance in law enforcement recruits [5]. Declines in upper-body strength and endurance, which have been shown for incumbent officers compared to cadets [15] and across different age groups [16,17], could negatively impact fence climbing tasks. In this study, the DS48+ group performed the CLF and SW slower than all other groups. The physiological characteristics important for obstacle climbing tasks may be maintained to some extent in deputy sheriffs post-academy, with notable changes only occurring after a longer period working in custody (i.e., 48 months or greater). Nevertheless, these results again emphasize the need for structured physical training for deputy sheriffs during their time in custody, as the change in work hours [15,46] and greater volume of low-intensity work [18,19,20] appear to negatively impact job-specific task performance. The implementation of consistent exercise could maintain performance in climbing tasks. Indeed, a four-foot wall climb was part of the obstacle course detailed by Rossomanno et al. [25]. 

In contrast to the other WSTB tasks, there were no significant differences between any of the groups in the BD. The BD in the WSTB simulates a victim drag or civilian rescue type scenario [5], which is often assessed in LEOs [3,4,5]. Lockie et al. [5] suggested that lower-body strength should contribute to this task, given the need for the individual to pick up the dummy from the ground and extend the legs to move to a standing position. However, absolute strength training is not often a focus of law enforcement academy training, with greater preference being given to aerobic training and calisthenics [5,12,47,48]. On the surface, it could be suggested that when compared to the REC, deputy sheriffs maintained the strength required to perform the BD during their tenure in custody. However, if the REC did not complete much absolute strength training during their academy [5,12,47,48], it could be that they are not that strong regardless of their performance in the BD. Taken further, that would mean the deputy sheriffs would not have a higher ceiling of strength to lose during their time in custody. However, these conclusions are speculative, and much further research is needed on the BD. This includes documenting relationships between lower-body strength and the BD, how absolute strength training could influence BD performance, and whether changes in absolute lower-body strength negatively impacts the BD. 

There are certain study limitations to this study that should be noted. This study was cross-sectional in nature, and the researchers were unable to compare the current data from the deputy sheriffs to when they were recruits graduating from academy. Future research should track deputy sheriff recruits from their time in academy to their time spent working in custody and in patrol positions to provide a more accurate picture of the effects of the occupation on fitness and job-specific task performance. This study utilized only one patrol school class, which featured 64 deputy sheriffs. This was a much smaller sample when compared to the REC, thus caution should be exercised when applying these results to other law enforcement populations. More research on a greater number of deputy sheriffs who have worked in custody is required, although the results from this study did reflect previous research, which found declines in the physical fitness of LEOs over time [15,16,17]. Although the REC would likely have completed the WSTB to the best of their ability so as to graduate academy, the motivation for the deputy sheriffs to perform the WSTB maximally may have been lower than the REC. This could have influenced the results from the deputy sheriff groups. Furthermore, the WSTB is only mandatory in the state of California in the USA [5,13]. Other states and countries may use different job-specific testing protocols, and these should be analyzed specifically for each agency, state, or country. Additionally, more general tests of fitness common to law enforcement populations (e.g., push-ups, sit-ups, jump tests, medicine ball throws, maximal running tests) [2,5,10,12,15,16,17,18,21,22,23,24,26,49,50,51] could be adopted in future research to track fitness changes relative to time spent working in custody for deputy sheriffs. These tests can be easier to administer while also representing the underlying physical qualities (e.g., strength, power, endurance, running speed) important for job tasks [52].

## 5. Conclusions

In conclusion, the results from this study indicated that deputy sheriffs who had worked in custody facilities for longer periods of time had poorer performance in WSTB tasks. Specifically, deputy sheriffs who had spent 48 months or greater were slower in the 99OC, CLF, SW, and 500R compared to recruits at the end of academy and deputy sheriffs who had worked for less time in custody. Deputy sheriffs who worked for 48 months or less were slower in the 500R compared to recruits. BD performance was not significantly different between recruits and deputy sheriffs, although that may be a function of a lack of strength training during academy, which would mean there is less absolute strength to decline when a deputy sheriff begins work in custody. These results suggest that structured physical training completed by the individual or provided by the LEA should be implemented to maintain fitness and job-specific task performance in deputy sheriffs. This is especially important for those deputy sheriffs who are leaving custody to begin working in a patrol position, as they will need to be able to perform the tasks that are assessed by the WSTB.

## Figures and Tables

**Table 1:** Descriptive data (mean ± SD) for age, height, body mass, and the Work Sample Test Battery (WSTB) tasks [99-yard obstacle course (99OC), body drag (BD), chain link fence climb (CLF), solid wall climb (SW), and 500-yard run (500R)] in recruits (REC), and deputy sheriffs who had spent ≤24 months (DS24), 25–47 months (DS2547), and ≥48 months (DS48+) working in custody.

Variables	REC (*n* = 253)	DS24 (*n* = 20)	DS2547 (*n* = 23)	DS48+ (*n* = 21)
Age (years)	26.69 ± 5.26	30.30 ± 6.36 *	30.30 ± 5.35 *	36.33 ± 6.26 *^,§,ɸ^
Height (m)	1.75 ± 0.10	1.74 ± 0.07	1.74 ± 0.09	1.71 ± 0.10
Body mass (kg)	79.69 ± 12.29	88.99 ± 13.68	85.46 ± 15.08	83.79 ± 19.35
99OC (sec)	18.49 ± 1.63	18.83 ± 1.60	19.13 ± 1.81	20.64 ± 2.25 *
BD (sec)	5.41 ± 3.19	4.64 ± 0.84	5.08 ± 0.68	6.17 ± 1.77
CLF (sec)	7.83 ± 1.20	7.60 ± 1.62	7.69 ± 1.89	9.78 ± 2.41 *^,§,ɸ^
SW (sec)	7.75 ± 1.37	7.63 ± 2.09	7.86 ± 1.82	9.83 ± 4.45 *^,§,ɸ^
500R (sec)	89.20 ± 7.99	100.75 ± 15.08 *	108.09 ± 38.80 *	113.19 ± 18.76 *

* Significantly (*p* < 0.05) different from REC; ^§^ Significantly (*p* < 0.05) different from DS24; ^ɸ^ Significantly (*p* < 0.05) different from DS2547.

**Table 2:** Pairwise effect size data between REC and deputy sheriffs who had spent ≤24 months (DS24), 25–47 months (DS2547), and ≥48 months (DS48+) working in custody for age, height, body mass, and the WSTB tasks (99OC, BD, CLF, SW, and 500R).

Variables	REC- DS24	REC-DS2547	REC- DS48+	DS24- DS2547	DS24- DS48+	DS2547- DS48+
Age	0.62 *	0.68 *	1.67 ^§^	<0.01	0.96 *	1.04 *
Height	0.12	0.11	0.40	<0.01	0.35	0.32
Body mass	0.72 *	0.42	0.25	0.25	0.31	0.10
99OC	0.21	0.37	1.09 *	0.18	0.93 *	0.74 *
BD	0.33	0.14	0.29	0.58	1.10 *	0.81 *
CLF	0.16	0.09	1.02 *	0.05	1.06 *	0.97 *
SW	0.07	0.07	0.63 *	0.12	0.63 *	0.58
500R	0.96 *	0.67	1.66 ^§^	0.25	0.73 *	0.17

* Moderate effect for the pairwise comparison; ^§^ Large effect for the pairwise comparison.
